# A perspective on large-scale simulation as an enabler for novel biorobotics applications

**DOI:** 10.3389/frobt.2023.1102286

**Published:** 2023-08-25

**Authors:** Emmanouil Angelidis

**Affiliations:** ^1^ Chair of Robotics, Artificial Intelligence and Embedded Systems, School of Informatics, Technical University of Munich, Munich, Germany; ^2^ Munich Research Center, Huawei Technologies Germany, Munich, Germany

**Keywords:** simulation, robotics, artificial intelligence, neuroscience, cloud computing

## Abstract

Our understanding of the complex mechanisms that power biological intelligence has been greatly enhanced through the explosive growth of large-scale neuroscience and robotics simulation tools that are used by the research community to perform previously infeasible experiments, such as the simulation of the neocortex’s circuitry. Nevertheless, simulation falls far from being directly applicable to biorobots due to the large discrepancy between the simulated and the real world. A possible solution for this problem is the further enhancement of existing simulation tools for robotics, AI and neuroscience with multi-physics capabilities. Previously infeasible or difficult to simulate scenarios, such as robots swimming on the water surface, interacting with soft materials, walking on granular materials etc., would be rendered possible within a multi-physics simulation environment designed for robotics. In combination with multi-physics simulation, large-scale simulation tools that integrate multiple simulation modules in a closed-loop manner help address fundamental questions around the organization of neural circuits and the interplay between the brain, body and environment. We analyze existing designs for large-scale simulation running on cloud and HPC infrastructure as well as their shortcomings. Based on this analysis we propose a next-gen modular architecture design based on multi-physics engines, that we believe would greatly benefit biorobotics and AI.

## 1 Introduction

Examining the complex interplay between an environment, a body and the brain, requires the use of multiple simulation tools, originating in neuroscience, AI and robotics. Closing the loop between such simulation tools has been a long-standing goal of the biorobotics community, with the target of being able to test hypotheses around the function of brain structures, sensorimotor loops, examine cognitive and behavioral hypotheses, but also to simply improve existing biorobots designs. Many of these simulation tools have been developed within the context of ambitious research projects such as the European Union’s the Human Brain Project (Markram et al., 2011), the Allen Human Brain Atlas developed by the Allen Institute for Brain Science, and the Japanese Brain/MINDS project (Okano et al., 2015). In order to develop such tools, a shift of perspective is necessary, from doing research in a specific field to instead an approach where the development of the tools themselves is the object of the research effort. These tools subsequently facilitate the researchers’ work by avoiding the need to do duplicate work, centralizing the output of multiple teams intoshared knowledge repositories and by advancing fields which might not be strictly related to the tools’ target community, with the overall impact of accelerated research output. An example of impact outside the target community’s goals, is the managing of big data related to neuroscience. Brain atlases might not be strictly related to neuroscience itself, but the knowledge acquired while building them indirectly contributes to other fields such as data science. By examining the most successful open-source projects in Machine Learning (ML) such as PyTorch (Paszke et al., 2019) and TensorFlow (Abadi et al., 2015) one can see that even though they are themselves are tools for other scientists to use, their influence has significantly advanced progress in ML.

The current state of simulation tools for neuroscience focuses on the solution of biophysical problems with various degrees of biological detail. The widely adopted NEST (Gewaltig and Diesmann, 2007), Brian (Stimberg et al., 2019), Neuron (Carnevale, 2007) and Nengo (Bekolay et al., 2014) simulators model the nervous system in low or high-level abstractions. In terms of the physical phenomena that are simulated, low-level models incorporate information from biophysical models and to the level of ion pumps and ion channels that regulate the membrane potential of neural cells. Point neuron models used in Spiking Neural Networks (SNN) employ integrate-and-fire (IF) types of neurons and are deprived of low-level biological information. Finally, other types of abstract models of cognition e.g., the Virtual Brain (Schirner et al., 2022) avoid the low-level complexity and instead focus on large-scale brain network activity, but can also be coupled with SNN models, enabling multi-scale simulation.

Robotics simulation is governed by open-source simulation tools such as Gazebo (Koenig and Howard, 2004), MuJoCo (Todorov et al., 2012), Webots (Michel, 2004), pybullet (Coumans and Bai, 2016). More recently multi-physics simulation tools such as Nvidia’s PhysX and Project Chrono (Tasora et al., 2016) have started gaining traction, adding robotics simulation features in their frameworks, but are still quite far from being widely adopted by the robotics research community, as analyzed in the report of usage of robotics simulators by (Collins et al., 2021). One limitation of existing robotics simulators is that they lack or minimally support features such as soft body simulation, fluid dynamics, plasticity etc., as analyzed in (Choi et al., 2021). This significantly reduces the range of biorobotics applications that can be simulated with current approaches, as many of the biorobots that have been developed implement concepts such as soft grasping, flexible mechanisms, interactions with fluids etc.

The neuroscience and robotics communities are often separated from each other, thus limiting the researchers’ ability to model complex phenomena in a more holistic approach that would model and most importantly couple multiple systems simultaneously. Large-scale simulation forces us to rethink the principles behind not only what physical phenomena to simulate, but also at what granularity and most importantly how different simulation tools can communicate with each other. This is particularly true for biorobotics, especially since biology, neuroscience and robotics each follow their own simulation paradigms and rarely interact in a systematic way. However, there is a trend of combining simulation tools accelerated by the progress in deep learning, giving us fine examples of how simulation tools for robotics can be integrated with other systems. A notable example is the integration of virtual simulation environments into deep learning frameworks such as OpenAI gym (Brockman et al., 2016). Such simulation environments that seamlessly integrate neural networks into robotics have been extremely successful in training robotic agents to perform various tasks, such as picking objects or locomotion tasks among others.

A core component of these simulation tools is that they are extremely modular, facilitating the integration of different frameworks into their core. This means that depending on the use case a different physics engine might be used, different 3D models format, as well as various DL frameworkse.g., PyTorch or TensorFlow. Such modular architectures enable the design of generic simulation tools around principles that are agnostic to the simulation engines that are chosen for a specific application. This approach is particularly suitable for biorobotics as the range of simulation tools varies significantly between different applications. As such, modular large-scale simulation designs that are agnostic to the specific simulation engines becomes a necessity for biorobotics research. We detail existing designs suitable for biorobotics, analyze their limitations and suggest a new paradigm based on multi-physics and simulation agnostic engine design.

## 2 The Neurorobotics Platform

The Neurorobotics Platform (NRP) (Falotico et al., 2017), developed within the context of the Human Brain Project, has formed as the basis of many fruitful experiments in neuroscience of rehabilitation (Allegra Mascaro et al., 2020), biologically-inspired robot control (Capolei et al., 2019; Angelidis et al., 2021), dynamic vision systems based on event-based cameras (Bornet et al., 2019; Kaiser et al., 2020), robot manipulation (Bing et al., 2021), pattern recognition (Galindo et al., 2020) among others. It is one of the few simulation engines that enable the interaction of SNNs with virtual robots, closing the loop between neuroscience and robotics simulation. It has a web-based user interface and python-based editors that enable the user to write minimal code that dictates the information exchange between virtual biorobots and the SNNs they are coupled with.

At the core of the NRP lies the Closed-Loop Engine (CLE), which from a simplified point of view is a python module that allows the exchange of data, synchronization and execution between the Gazebo robotics simulator and various neural backends (NEST, Nengo) and neuromorphic hardware such as SpiNNaker (Furber et al., 2014) and Loihi (Davies et al., 2018). At the same time it is running on High Performance Computing (HPC) infrastructure inside docker containers that facilitate deployment. ROS 1 (Quigley et al., 2009) is used as the middleware that packs and publishes the data that needs to be exchanged between the different simulation engines.

Even though the design of the NRP and its CLE are in principle quite generic, in practice its implementation is tied down to the specifics of the engines that it supports, making it cumbersome to add new simulation engines e.g., for robotics simulation, such as MuJoCo, PhysX, Webots etc. As there is no “one-engine-that-fits-all” in robotics, as different applications and communities use different tools, the NRP ‘s support for only a subset of the available simulators has limited its wide adoption by the biorobotics community. Furthermore, the use of ROS 1 as middleware leads to non-deterministic simulations that are difficult to reproduce. Tied to some of the limitations of Gazebo, it does not provide photorealistic rendering, easy to use 3D modelling tools, or support for large-scale environments. It should be noted that the newest versions of Gazebo, termed Gazebo Ignition have been designed around an architecture that enables the distribution of the physics simulation into different computational nodes, facilitating large-scale environment simulation. Another difficulty is the simulation of multiple robots in parallel, especially ones that have many degrees of freedom, such as humanoid robots. The design limitations of the first version of the NRP led to the redesign of its architecture around a more modular design, but at the time of the writing of this publication, the new architecture has not been released. We discuss a potential design for biorobotics applications that overcomes some of the aforementioned limitations.

## 3 A paradigm for large-scale simulation design

Simulation is in some cases, such as training locomotion controllers based on Reinforcement Learning (RL) methods, the only viable approach as the generation of large amounts of training data is not possible on the real robots. Furthermore, novel robot designs have to be tested in simulation before a physical counterpart is created, and safety considerations prevent certain algorithms from being deployed directly on the hardware. In RL scenarios virtual robots interacting with virtual environments generate vast amounts of data that comes from the simulation, and which are in turn fed into an AI-based model. These simulations run in parallel, often in thousands of instances that share data between them. After an algorithm has been developed in simulation it often tried on the real robot, albeit in most cases with limited success, at least during the initial phases of the transferred policies.

Several factors contribute to the simulation-to-real (sim2real) gap (Salvato et al., 2021). Imperfect simulated sensors and controllers, uncertainty in the parametrization of dynamics data, simplified physics models, difficulty to estimate physical parameters such as viscosity, friction etc., are some of the sources of discrepancy between simulation and reality. Strategies which have been employed to close the sim2real gap, include the development of more realistic simulations, accurate sensor models, domain randomization, addition of photorealism in simulations, especially in workflows that employ simulated visual data. It should be noted that each of these strategies come with its own cost in terms of computational load and difficulty of integration into a fully-fledged system that encompasses all of them.

We can identify improvements in four distinct fronts can be identified, which would help close the sim2real gap, leading to the development of a next-generation of intelligent biorobots.

1. Coupling between different components and engines.
2. Scalability around cloud infrastructure.
3. Support for new types of hardware.
4. Photorealistic rendering.

**FIGURE 1:**
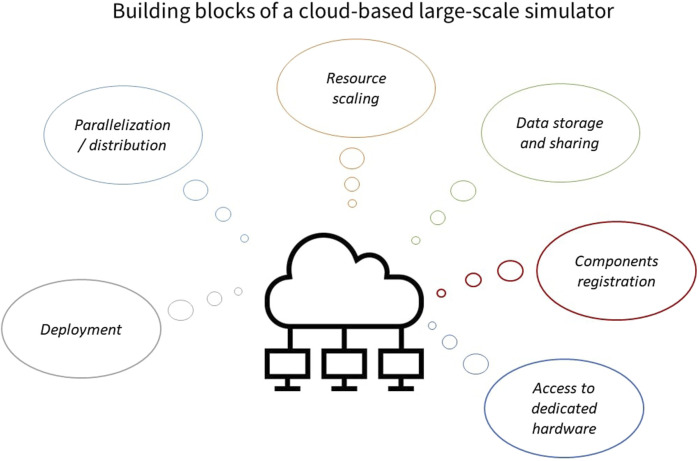
A large-scale simulation design for the closing of the loop between AI and robotics. In this design the core of a simulation framework provides interfaces that allow for the easy registration of external modules that provide simulation services. The core manages data exchange, synchronization and execution of the different simulation components. Cloud services provide facilities like data storage and resource scaling. As examples existing robotics simulators like Gazebo, Webots, MuJoCo, AirSim, bullet, CoppeliaSim can be easily coupled with AI frameworks such as TensorFlow, PyTorch and Caffe as well as neuroscience-based simulators like Nest, Nengo and neuromorphic backends such as BrainScaleS, SpiNNaker, Loihi. This design goes beyond the purpose of connecting AI based models with robotics simulation and can be generalized to other components that provide more specialized forms of simulation. The core advantage is that the integration of new components into this design is as simple as providing data interfaces that generate and consume data, keeping data exchange between simulators to a minimum.

In more detail, a systems design approach where coupling between components of a simulation engine agnostic to the specifics of the components is necessary (Figure 1). Such a design is based on clear interfaces and abstractions that facilitate the exchange of data. This is achievable e.g., through the use of middleware and serialization software such as the Robot Operating System (ROS1/ROS2), google-protobuffs etc. Through such mechanisms a user can define abstractions at the right level that enable the exchange of a minimal set of data between the different components. As an example, exchanging data from SNNs would need a minimal set of arrays of spike-times along with neuron IDs. For robotics, the robots’ joints, links poses, and sensory data might suffice. Coupling is then reduced to asynchronization and data exchange step where through interfaces, data is being streamed from one component to the other that requests it. It is implied that there is an initial setup step necessary where each component must declare its own interface of data that it consumes as well as data that it produces.

**FIGURE 2:**
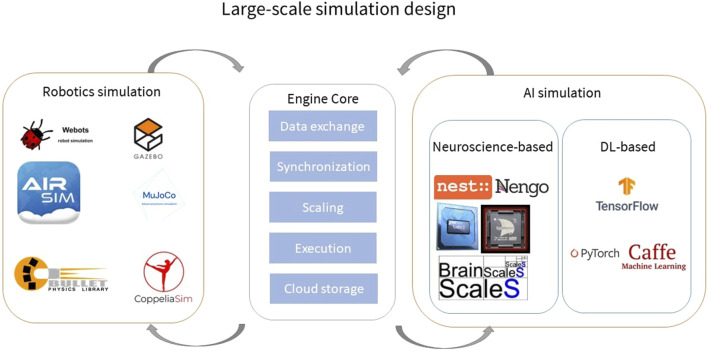
Building blocks of a cloud-based large-scale simulator. The advantages of the cloud-based architecture revolve around the automatic deployment of simulators, parallelization of simulations into multi-CPU and multi-GPU servers, and distribution into multiple computational nodes via automated resource scaling services. Data sharing and storage, a necessity for the exchange of information between different instances of a simulation is a native scenario for cloud architectures. Registering components of a simulation service can be done easily with minimum code provided through web-interfaces. Finally, access to more exotic types of hardware that are difficult to acquire, such as TPUs, neuromorphic hardware can be provided through the cloud infrastructure.

The second point of scalability is where cloud computing and HPC infrastructure come to play. Considerations of software deployment, workload balancing, parallelization, launching of multiple simulation instances in parallel, synchronization and exchange of data are a natural application scenario for HPC and cloud computing. In a cloud-based architecture (Figure 2) where a system as the one that we described in the previous paragraph has been deployed, a user can programmatically register new containerized easy-to-deploy components that adhere to well-defined data exchange interfaces. These components could be anything from simulation engines to complex databases and knowledge graphs, ideally letting the user interact through a browser window without the need to develop on their own servers. Dedicated services facilitate the sharing and storage of large amounts of data, such as simulation states, episodic returns, brain connectivity atlases etc. Parallelization and distribution of simulations, even though it is not easily achievable in a simulator agnostic way, would be one of the main benefits. On-demand scalable computational power is one of the features of cloud-based architectures, which can be combined with access to specialized hardware such as Tensor Processing Units (TPUs), neuromorphic backends, and multi-GPUs. Given all these building blocks, the user would need to specify the simulation models that they would like to simulate, giving them a large-scale simulation engine, leaving only the testing of scientific hypotheses to the user.

The third component of large-scale simulation is the support for new types of hardware, especially neuromorphic ones that help close the loop between neuroscience and robotics. BrainScales, SpiNNaker, Loihi are all deployed on the cloud and access is given to users that can run their own experiments. However, integrating these novel types of hardware into engines that provide physical simulation poses a challenge. Getting them to run in a closed loop manner that would exchange data between them, and physical simulators used in robotics is no easy feat. The weight falls on the device and hardware manufacturers to enhance them with the ability to step them explicitly and performing I/O operations without harming their real-time or even faster than real-time performance capabilities. This, accompanied with features such as energy benchmarking on the chip can significantly reduce the access to novel types of hardware.

Finally, as the generation of training data in simulation is a necessity for intelligent biorobots, the enhancement of existing simulators with photorealistic capabilities becomes essential. The current generation of robotics simulators (Gazebo, MuJoCo, Webots, pybullet, CopelliaSim etc.) focus on getting the physics right, and have limited photorealistic rendering capabilities. This means that data generation for visual perception tasks in simulation are not realistic enough, and is thus an important contributor to the sim2real gap. On the other hand, the latest generation of game engines such as the Unreal Engine 5, Unity and O3DE have excellent photorealistic rendering capabilities and can be used as the visualization part of large-scale simulation. Furthermore, they have a rich ecosystem of 3D modelling pipelines. There is a limitation though, that games do not need the same degree of physical realism as robotics applications, hence the physics engines that power them are not accurate enough. Hence, the combination of games engines that offer photorealistic rendering with high-accuracy physics engines becomes a necessity for intelligent robotics simulation. Next, we discuss how multi-physics and unified physics engines can open new paths in biorobotics simulation.

## 4 Multi-physis as the enabler of novel applications

Quoting Auke Ijspeert from 2014 (Ijspeert, 2014):

“Robots are better than numerical simulations when real (as opposed to simulated) physics is important. This is often the case with locomotion that involves complex physical interactions with the environment: swimming in water, crawling on sand, and walking on mud or gravel. Mechanical simulations are now very good at computing the dynamics of articulated rigid bodies but are less good at correctly simulating compliant structures and interaction forces with a complex environment.”

This assessment is a good explanation of the sim2real gap, and most importantly it highlights the limitations of physical simulation for robotics. Especially complex real-world scenarios where a robot has to interact with previously unseen environments, such as climbing stairs, environments with changing friction, interactions with fluids, cloths and soft bodies among others are notoriously difficult to simulate. Historically physics engines for robotics have focused on the solution of articulated rigid body dynamics problems, leaving fluids, soft bodies, muscle, cloth simulation out of their features list (Angelidis et al., 2022). This holds true for most of the widely used simulators in the robotics and biorobotics community as assessed by Collins (Collins et al., 2021). For those engines that do provide some form of coupling of their rigid body dynamics solver with some other form of physical simulation e.g., finite elements for soft body dynamics, the coupling is done externally via exchange of forces/torques, positions.

Even though external (also termed loosely) coupling has proven sufficient for a wide category of applications it faces certain challenges. Firstly, there is no guarantee that different solvers will run on the same hardware, it is common for fluid dynamics solvers to run on the GPU but a basic CPU implementation of rigid body dynamics is enough for many robotics applications. Running solvers on different hardware raises concerns over the need to constantly perform costly Input/Output (I/O) operations that are detrimental for real time performance. Secondly, external coupling raises concerns over the stability of the simulation. There is no mathematical guarantee that running two solvers separately and coupling them means that the common solution has converged numerically.

Recently, an approach of so-called unified physics solvers has emerged which revolves around the idea of simulating the dynamics of bodies via constraints that are resolved on the position level (Bender et al., 2014), as opposed to the computation of forces or impulses. The common ground of these approaches is to represent deformable objects as a set of interconnected nodes or particles that are connected with constraints e.g., spring, friction, collision constraints. Each particle has a set of attributes such as position, velocity and mass. The aforementioned constraints can correspond to geometrical, dynamical i.e., velocity constraints or even represent constitutive material laws e.g., the stress-strain relationship in soft body simulations with finite elements. The advantage of simulating all bodies as constraints is that the coupling between different phenomena is implicit in the mathematical formulation, delegating the dynamics computations to a constraint solver plus a time-integration scheme. In more detail, during the constraint resolution step, the position of the particles is updated in a manner that satisfies one or multiple constraints. Typically, the constraints at the position level are provided by a constraint function that estimates whether the constraint is satisfied e.g., the distance between particles is at a desired value. When the constraint is not satisfied, the gradient of the constraint function with respect to the positions of the particles is computed and subsequently the particles’ position is corrected towards the gradient. This process is repeated up to a desired convergence. By defining appropriate constraints one can simulate fluids, cloths, rigid/soft bodies, muscles, contact problems, ropes, 1-D rods etc. The most popular of these methods is Extended Position Based Dynamics (XPBD) (Müller et al., 2020), developed by the Nvidia Physics team and incorporated into the Nvidia PhysX and FleX (Macklin et al., 2014) solvers. Another approach is termed Projective Dynamics (Bouaziz et al., 2014) and its main idea is to combine the XPBD approach with ideas from nodal finite elements into a unified solver. All these approaches have the potential to serve as the unifying framework for realistic simulations of biorobots and their complex interactions with their environment.

Thus, in combination with large-scale modular closed-loop simulations for biorobotics, unified physics open a new wide range of research directions and an excellent opportunity to close the sim2real gap. More precisely we can envision and suggest research directions in locomotion on complex terrains e.g., sand, gravel, fluids, highly-viscous and elastic surfaces that would greatly improve the mobility of biorobots and their adaptivity in previously unseen environments. Locomotion modes which are notoriously difficult to simulate e.g., climbing on walls or trees, swimming on free surfaces can become feasible simulation scenarios. However, it should be noted that the accuracy of physics simulation based on constrained based solvers is computationally intensive when accuracy is a requirement and for real-time applications might not be close enough to reality. As such, integrating them into ML-based methods and enhancing them with techniques such as domain randomization is a viable approach that would help further minimize the sim2real gap.

Lastly, combining all the building blocks that we discussed above, photorealistic rendering, integration into AI-based frameworks, unified physics in combination with cloud-based infrastructure would enable novel directions in neuroscience, robotics and AI simulation.

## 5 Conclusion

We discussed two key enablers of a new generation of simulations, large-scale modular cloud-native simulation design, and multi-physics capabilities for increased realism. As the trend of designing, evaluating and testing algorithms and control methods before deploying them on the physical hardware seems to be inevitable, simulation tools that adhere to the aforementioned principles are becoming a necessity. Closing the sim2real gap via improved simulation methods on top of existing techniques is becoming a key enabler of intelligent robots towards the goal of reaching human or superhuman performance.

## Data Availability

The original contributions presented in the study are included in the article/supplementary material, further inquiries can be directed to the corresponding author.

## References

[B1] Abadi M. Agarwal A. Paul B. Brevdo E. Chen Z. Craig C. (2015). TensorFlow: Large-Scale machine learning on heterogeneous systems.

[B2] Allegra Mascaro A. L. Falotico E. Petkoski S. Pasquini M. Vannucci L. Tort-Colet N. (2020). Experimental and computational study on motor control and recovery after stroke: toward a constructive loop between experimental and virtual embodied neuroscience. Front. Syst. Neurosci. 14, 31. 10.3389/fnsys.2020.00031 32733210 PMC7359878

[B3] Angelidis E. Bender J. Arreguit J. Gleim L. Wang W. Axenie C. (2022). “Gazebo fluids: SPH-Based simulation of fluid interaction with articulated rigid body dynamics,” in 2022 IEEE/RSJ International Conference on Intelligent Robots and Systems (IROS), Kyoto, Japan, 23-27 October 2022. 11238–11245. 10.1109/IROS47612.2022.9982036

[B4] Angelidis E. Buchholz E. Arreguit J. Rougé A. Stewart T. Arnim A. von (2021). A spiking central pattern generator for the control of a simulated lamprey robot running on SpiNNaker and Loihi neuromorphic boards. Neuromorphic Comput. Eng. 1, 014005. 10.1088/2634-4386/ac1b76

[B5] Bekolay T. Bergstra J. Hunsberger E. DeWolf T. Stewart T. Rasmussen D. (2014). Nengo: A Python tool for building large-scale functional brain models. Front. Neuroinformatics 7, 48–13. 10.3389/fninf.2013.00048 PMC3880998 24431999

[B6] Bender J. Koschier D. Charrier P. Weber D. (2014). Position-based simulation of continuous materials. Comput. Graph. 44, 1–10. 10.1016/j.cag.2014.07.004

[B7] Bing Z. Brucker M. Morin F. O. Li R. Su X. Huang K. (2021). Complex robotic manipulation via graph-based hindsight goal generation. IEEE Trans. Neural Netw. Learn. Syst. 33, 7863–7876. 10.1109/TNNLS.2021.3088947 34181552

[B8] Bornet A. Kaiser J. Kroner A. Falotico E. Ambrosano A. Cantero K. (2019). Running large-scale simulations on the Neurorobotics Platform to understand vision–the case of visual crowding. Front. Neurorobotics 13, 33. 10.3389/fnbot.2019.00033 PMC6549494 31191291

[B9] Brockman G. Cheung V. Pettersson L. Schneider J. Schulman J. Tang J. (2016). OpenAI gym.

[B10] Capolei M. C. Angelidis E. Falotico E. Lund H. H. Tolu S. (2019). A biomimetic control method increases the adaptability of a humanoid robot acting in a dynamic environment. Front. Neurorobotics 70, 70. 10.3389/fnbot.2019.00070 PMC6722230 31555117

[B11] Carnevale T. (2007). Neuron simulation environment. Scholarpedia 2, 1378. 10.4249/scholarpedia.1378

[B12] Choi H. Crump C. Duriez C. Elmquist A. Hager G. Han D. (2021). On the use of simulation in robotics: opportunities, challenges, and suggestions for moving forward. Proc. Natl. Acad. Sci. 118, e1907856118. 10.1073/pnas.1907856118 33323524 PMC7817170

[B13] Collins J. Chand S. Vanderkop A. Howard D. (2021). A review of physics simulators for robotic applications. IEEE Access 9, 51416–51431. 10.1109/ACCESS.2021.3068769

[B14] Coumans E. Bai Y. (2016). Pybullet, a python module for physics simulation for games, robotics and machine learning.

[B15] Davies M. Srinivasa N. Lin T.-H. Chinya G. Cao Y. Choday S. H. (2018). Loihi: A neuromorphic manycore processor with on-chip learning. IEEE Micro 38, 82–99. 10.1109/MM.2018.112130359

[B16] Falotico E. Vannucci L. Ambrosano A. Albanese U. Ulbrich S. Vasquez Tieck J. C. (2017). Connecting artificial brains to robots in a comprehensive simulation framework: the Neurorobotics Platform. Front. Neurorobotics 11, 2. 10.3389/fnbot.2017.00002 PMC5263131 28179882

[B17] Furber S. B. Galluppi F. Temple S. Plana L. A. (2014). The SpiNNaker project. Proc. IEEE 102, 652–665. 10.1109/JPROC.2014.2304638

[B18] Galindo S. E. Toharia P. Robles Ó. D. Ros E. Pastor L. Garrido J. A. (2020). Simulation, visualization and analysis tools for pattern recognition assessment with spiking neuronal networks. Neurocomputing 400, 309–321. 10.1016/j.neucom.2020.02.114

[B19] Gewaltig M.-O. Diesmann M. (2007). NEST (NEural simulation tool). Scholarpedia 2, 1430. 10.4249/scholarpedia.1430

[B20] Ijspeert A. J. (2014). Biorobotics: using robots to emulate and investigate agile locomotion. Science 346, 196–203. 10.1126/science.1254486 25301621

[B21] Kaiser J. Friedrich A. Tieck J. C. V. Reichard D. Roennau A. Neftci E. (2020). “Embodied neuromorphic vision with continuous random backpropagation,” in 2020 8th IEEE RAS/EMBS International Conference for Biomedical Robotics and Biomechatronics (BioRob), New York, NY, USA, 29 November 2020 - 01 December 2020. 1202–1209. 10.1109/BioRob49111.2020.9224330

[B22] Koenig N. Howard A. (2004). “Design and use paradigms for Gazebo, an open-source multi-robot simulator,” in 2004 IEEE/RSJ International Conference on Intelligent Robots and Systems (IROS) (IEEE Cat. No.04CH37566), Sendai, Japan, 28 September 2004 - 02 October 2004, 2149–2154. 10.1109/IROS.2004.1389727

[B23] Macklin M. Müller M. Chentanez N. Kim T.-Y.,(2014). Unified particle physics for real-time applications. ACM Trans. Graph. 33 (153), 1–12. 10.1145/2601097.2601152

[B24] Markram H. Meier K. Lippert T. Grillner S. Frackowiak R. Dehaene S. (2011). Introducing the human brain project. Procedia Comput. Sci., Proc. 2nd Eur. Future Technol. Conf. Exhib. 7, 39–42. 10.1016/j.procs.2011.12.015

[B25] Michel O. (2004). Cyberbotics ltd. Webots™: professional mobile robot simulation. Int. J. Adv. Robot. Syst. 1, 5. 10.5772/5618

[B26] Müller M. Macklin M. Chentanez N. Jeschke S. Kim T.-Y. (2020). Detailed rigid body simulation with extended position based dynamics. Comput. Graph. Forum 39, 101–112. 10.1111/cgf.14105

[B27] Okano H. Miyawaki A. Kasai K. (2015). Brain/MINDS: brain-mapping project in Japan. Philos. Trans. R. Soc. B Biol. Sci. 370, 20140310. 10.1098/rstb.2014.0310 PMC4387516 25823872

[B28] Paszke A. Gross S. Massa F. Lerer A. Bradbury J. Chanan G. (2019). PyTorch: An imperative style. High-Performance Deep Learning Library. 10.48550/arXiv.1912.01703

[B29] Quigley M. Conley K. Gerkey B. Faust J. Foote T. Leibs J. (2009). Ros: An open-source robot operating system. ICRA Workshop on Open Source Software.

[B30] Salvato E. Fenu G. Medvet E. Pellegrino F. A. (2021). Crossing the reality gap: A survey on sim-to-real transferability of robot controllers in reinforcement learning. IEEE Access 9, 153171–153187. 10.1109/ACCESS.2021.3126658

[B31] Schirner M. Domide L. Perdikis D. Triebkorn P. Stefanovski L. Pai R. (2022). Brain simulation as a cloud service: the Virtual Brain on EBRAINS. NeuroImage 251, 118973. 10.1016/j.neuroimage.2022.118973 35131433

[B32] Stimberg M. Brette R. Goodman D. F. (2019). Brian 2, an intuitive and efficient neural simulator. eLife 8, e47314. 10.7554/eLife.47314 31429824 PMC6786860

[B33] Tasora A. Serban R. Mazhar H. Pazouki A. Melanz D. Fleischmann J. (2016). “Chrono: an open source multi-physics dynamics engine,” in High performance computing in science and engineering, lecture notes in computer science. Editors Kozubek T. Blaheta R. Šístek J. Rozložník M. Čermák M. (Cham: Springer International Publishing), 19–49. 10.1007/978-3-319-40361-8_2

[B34] Todorov E. Erez T. Tassa Y. (2012). “Mujoco: A physics engine for model-based control,” in 2012 IEEE/RSJ International Conference on Intelligent Robots and Systems, Vilamoura-Algarve, Portugal, 07-12 October 2012, 5026–5033.

